# Editorial: Extracellular Nucleotides in Lymphocyte Function

**DOI:** 10.3389/fcell.2022.892303

**Published:** 2022-03-23

**Authors:** Henrique Borges da Silva, Maria Regina D’Imperio Lima, Luiz Eduardo Baggio Savio

**Affiliations:** ^1^ Department of Immunology, Mayo Clinic, Scottsdale, AZ, United States; ^2^ Department of Immunology, Instituto de Ciencias Biomedicas, Universidade de Sao Paulo, Sao Paulo, Brazil; ^3^ Laboratory of Immunophysiology, Instituto de Biofisica Carlos Chagas Filho, Universidade Federal do Rio de Janeiro, Rio de Janeiro, Brazil

**Keywords:** lymphocyte, P2RX7, ectonucleotidase, danger signal, nucleotides, solid tumor, immune response

The optimal function of lymphocytes (e.g., T and B cells) requires the sensing of a plethora of signals, many of them coming from the extracellular environment. Many of these signals are actively produced and secreted by immune and non-immune cells, e.g., cytokines (Nolz and Richer, 2020). In addition, the passive release of intracellular metabolites plays an important role in regulating lymphocyte function and homeostasis. A prominent class of these metabolites comprises extracellular nucleotides, such as ATP. Release of extracellular ATP (eATP) is often understood as a “danger signal,” i.e., a factor that typifies a situation of threat to the host in face of a disturbance: infections, cancer, or autoimmunity (Di Virgilio et al., 2017). The signaling role of eATP is well-defined for innate immune cells as a crucial activator of the NLRP3 inflammasome (Mariathasan et al., 2006). In the last couple of decades, however, a growing body of work has highlighted the importance of signaling through eATP (and other extracellular nucleotides) for lymphocyte function. In this Special Issue, we present a mix of literature reviews and primary research shedding light on the latest advances in our understanding of this important subject.

Release of eATP occurs at particularly high levels inside solid tumors, constituting a fundamental part of the tumor microenvironment (TME). Despite this prevalence, only recently the role of eATP signaling in regulating antitumor lymphocyte function has been done, especially focusing on T cells. The eATP receptor with the most well-defined immune function is the low-affinity P2RX7 ion channel (Di Virgilio et al., 2017). P2RX7 is expressed in antigen-experienced T cells, including those infiltrating solid tumors (Di Virgilio et al., 2017). Curiously, both the wild-type and mutant forms of P2RX7 are expressed at high levels by tumor cells themselves, with a seemingly pro-tumorigenic role. The concomitant expression of this receptor by tumor and T cells in the same TME makes it hard to predict and interpret how manipulation of this signaling pathway would influence the antitumoral T cell responses. The Perspective article by Grassi and Conti provides a comprehensive overview of the recent research and potential future directions on this subject. The association between P2RX7 and cancer is not limited to solid tumors, however. Perhaps linked to its expression in lymphocytes, P2RX7 is found to be expressed in certain hematologic tumor cells, such as leukemic cells. In fact, some of first connections between P2RX7 and oncogenesis were found on lymphocytic leukemia (Wiley and Dubyak, 1989). The Mini Review by De Marchi et al. shows the previous and recent discoveries on this subject and speculates about unanswered questions.

P2RX7 is expressed in many distinct lymphocyte subsets, and recent research has unveiled how this receptor regulates these subsets (Di Virgilio et al., 2017). This is especially true for “helper” CD4^+^ T cells: it is now understood that P2RX7 promotes the cell death of regulatory T cells (Treg) and follicular helper T cells (Tfh) (Taylor et al., 2007; Proietti et al., 2014). In contrast, P2RX7 has a positive role for type 1 helper T cells (Th1) (Salles et al., 2017). Less is known about Th17 cells, a subset specialized in the production of the IL-17 cytokine and subsequent activation of antibacterial responses (Korn et al., 2009). The Article from Yang et al. describes how the expression of P2RX7 in CD4^+^ T cells (and in the antigen-presenting dendritic cells) can increase the Th17 differentiation potential of CD4^+^ T cells.

Often after eATP release, ectonucleotidases present either in the extracellular environment or attached to cellular plasma membranes (e.g., CD39, CD73) can cleave eATP into its most common metabolites: eADP and the ultimate product, extracellular Adenosine. Opposite to eATP, Adenosine is typically understood as an immunomodulatory signal, being associated with T cell dysfunction in the context of solid tumors, which display high levels of Adenosine (Moesta et al., 2020). Less is understood about how ectonucleotidases and Adenosine influence lymphocyte function in the context of immune responses in solid, non-lymphoid organs. The Mini Review by Savio et al. addresses the previous and recent discoveries on how ectonucleotidases affect T cell responses in two specific organs, the gut and the liver, focusing on immune responses to autoimmune diseases in these organs.

Expression of ectonucleotidases is a common feature of cytotoxic CD8^+^ T cells, such as CD39 (which cleaves eATP into eADP) and CD73, which leads to the production of Adenosine (Moesta et al., 2020). As exposed above, the presence of these receptors is often linked to dysfunction of CD8^+^ T cells. The Article from Briceño et al. offers additional evidence on this direction, showing that expression of CD73 by CD8^+^ T cells leads to decreased mitochondrial metabolism and associated lower ability to infiltrate and control melanoma tumors in mice, with simultaneous avoidance of acquisition of an exhaustion phenotype. It is less clear, however, how the immunomodulatory role of ectonucleotidases affects CD8^+^ T cell responses to antigen stimulation that ceases quickly (such as an acute infection). The Article from Rosemblatt et al. suggests an interesting scenario: while, during the antigen stimulation, CD73 expression leads to reduced CD8^+^ T cell viability, in the situation of homeostasis (which would occur, for example, after antigen is gone), CD73 favors CD8^+^ T cell survival. These results perhaps agree with the fact that some memory CD8^+^ T cells express CD73 (Fang et al., 2021). It will be necessary to understand how CD73 promotes such distinct outcomes in these two different scenarios in future studies.

In summary, this Issue offers a comprehensive overview of how this sub-field stands nowadays. The discussions provided by these studies will be important, in our opinion, to shape future research aiming to better define how extracellular nucleotides affect lymphocyte function and homeostasis. In addition, they serve to highlight that eATP and Adenosine are much more than mere “danger-associated” signals as previously defined.
